# Polyamine Sharing between Tubulin Dimers Favours Microtubule Nucleation and Elongation via Facilitated Diffusion

**DOI:** 10.1371/journal.pcbi.1000255

**Published:** 2009-01-02

**Authors:** Alain Mechulam, Konstantin G. Chernov, Elodie Mucher, Loic Hamon, Patrick A. Curmi, David Pastré

**Affiliations:** 1 Laboratoire Structure-Activité des Biomolécules Normales et Pathologiques, Université Evry-Val d'Essonne, Evry, France; 2 INSERM, U829, Evry, France; 3 Institute of Protein Research, Russian Academy of Sciences, Pushchino, Moscow Region, Russia; INSERM : E0229, CRLCC Val d'Aurelle - Paul Lamarque, France

## Abstract

We suggest for the first time that the action of multivalent cations on microtubule dynamics can result from facilitated diffusion of GTP-tubulin to the microtubule ends. Facilitated diffusion can promote microtubule assembly, because, upon encountering a growing nucleus or the microtubule wall, random GTP-tubulin sliding on their surfaces will increase the probability of association to the target sites (*nucleation sites or MT ends*). This is an original explanation for understanding the apparent discrepancy between the high rate of microtubule elongation and the low rate of tubulin association at the microtubule ends in the viscous cytoplasm. The mechanism of facilitated diffusion requires an attraction force between two tubulins, which can result from the sharing of multivalent counterions. Natural polyamines (*putrescine, spermidine, and spermine*) are present in all living cells and are potent agents to trigger tubulin self-attraction. By using an analytical model, we analyze the implication of facilitated diffusion mediated by polyamines on nucleation and elongation of microtubules. *In vitro* experiments using pure tubulin indicate that the promotion of microtubule assembly by polyamines is typical of facilitated diffusion. The results presented here show that polyamines can be of particular importance for the regulation of the microtubule network *in vivo* and provide the basis for further investigations into the effects of facilitated diffusion on cytoskeleton dynamics.

## Author Summary

Interactions between biomolecules (DNA, proteins, sugar, etc.) represent the link between genome information and function of living organisms. For effective competition between organisms and adaptation to environmental changes, these interactions have to take place at very high rates. As such interactions require successive associations and dissociations between biomolecules, most of the time could be spent in between interactions when biomolecules diffuse freely in the intracellular space until the next interaction occurs. To reduce this waste of time, cells have developed adaptive mechanisms. First, the concentration of biomolecules in the intracellular medium is very high, which increases their frequency of interactions. Second, the possibility for biomolecules to diffuse along linear polymers, a process named “facilitated diffusion,” increases the probability for biomolecules to find their targets. This mechanism was first described to understand how DNA-binding proteins could find their specific targets among thousands of putative binding sites. We show here that facilitated diffusion could also play a significant role in promoting the assembly of cytoskeleton proteins that are involved in critical cell functions (e.g., division or neuron architecture). Alteration of this mechanism may be of particular interest for cancer and neuropathologies.

## Introduction

Microtubules (*MTs*) are αβ-tubulin hollow polymers that perform major organizational tasks in eukaryotic cells [1]. These structures, which contribute to the rigidity and structural integrity of the cells, are implicated in motor-driven intracellular traffic of organelles and vesicles, in the formation of the mitotic spindle and in cell migration and motility. At least two physical parameters counteract microtubule assembly in cells: (i) their highly negatively charged surface due to the negative charge of the αβ-tubulin heterodimer itself (20–30 e^−^) [2]–[4], which induces an electrostatic self-repulsion between tubulin molecules [5],[6], (ii) the viscosity of the cytoplasm which slows down tubulin diffusion (about *5.10^−12^ m^2^/s* as measured in sea urchin extracts [7]). Consequently, due to the requirement of a proper orientation of tubulin to associate to MT ends [8],[9], the flow of tubulin arriving directly to microtubule ends by 3D diffusion is critical to sustain the rapid elongation of microtubules observed in cells (>10 µm/s [10]). We propose here that facilitated diffusion may significantly enhance the association of tubulin to growing nuclei or microtubules. The mechanism of facilitated diffusion was already introduced to understand how DNA-binding proteins can find their target sites by sliding and hopping along DNA molecules [11],[12]. However facilitated diffusion can also favour tubulin association to nucleus and microtubule ends provided that an attraction exists between tubulins and microtubules or nucleus to enable sliding. This attraction force can be triggered by the presence of small multivalent cations that are shared between tubulin dimers. Among small cations, polyamines such as divalent putrescine, trivalent spermidine and tetravalent spermine are reasonable candidates due to their high concentrations in all cells [13],[14]. Surprisingly, despite the potential influence of polyamines on tubulin dynamics in cells [15],[16], only a few studies have addressed the mechanisms by which they act on microtubule assembly. The enhancement of the polymerization rate observed *in vitro* was generally attributed to the neutralization of the C-terminal tails of tubulin [2],[17],[18]. In addition to this effect, facilitated diffusion can also explain how multivalent cations actually act on the different aspects of microtubule assembly, notably nucleation and elongation.

In this paper we thus decipher, both theoretically and experimentally, if facilitated diffusion can interfere with microtubule dynamics. Taking advantage of the recent advances in the description of the electrostatic properties of tubulin [3],[4],[19], we show that the attraction force between highly anionic tubulins, mandatory for facilitated diffusion, can indeed occur due to the presence of polyamines. We then develop an analytical model to describe the influence of facilitated diffusion on microtubule assembly. Considering an attraction between tubulin dimers, we propose that facilitated diffusion can act on MT dynamics via two different ways: (i) a longer residency time of tubulin onto a growing nucleus which favours association, (ii) a facilitated elongation thanks to sliding of free tubulin to the MT ends. A series of *in vitro* experiments is conducted to test the model through the analysis of light scattering curves, sedimentation assays and high resolution AFM imaging of MTs. Finally, to approach more physiological conditions, we show some preliminary results for MAPs-tubulin.

## Results

### Tubulin:Tubulin Attraction Mediated by Polyamine Sharing

**Figure 1:**
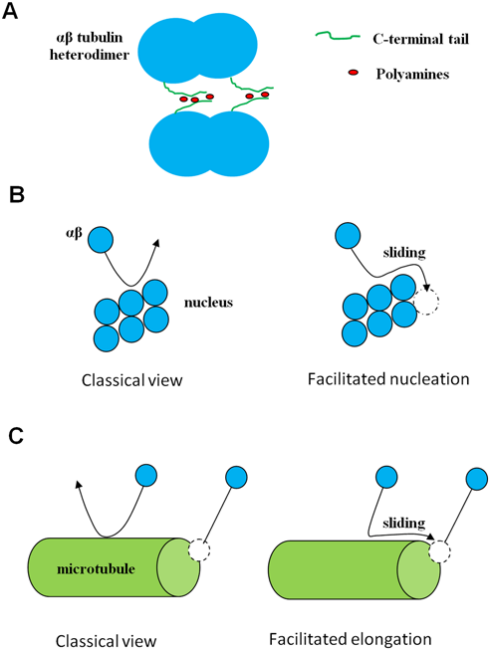
Schematic representation of the mechanism of facilitated diffusion. (A) Attraction between two tubulin heterodimers mediated by polyamines. The attraction is mediated by the two C-terminal tails of one tubulin dimer, which, for entropic reasons (*separation distance between the two C-termini tails on a tubulin dimer*), are likely to share polyamines with two C-terminal tails of another tubulin dimer. (B) In the absence of an attraction force, many collisions between tubulin and nucleus do not result in association, whereas, in the presence of an attraction force, both facilitated diffusion via sliding and an increase of the interaction lifetime favour association to the growing nucleus. (C) In the presence of an attraction force, sliding of tubulin along the cylindrical surface of MT favours tubulin association to the MT ends.

Multivalent cations like polyamines can induce an electrostatic attraction between two highly negatively charged surfaces. The theoretical treatment of this force has been the subject of extensive studies [20]–[23] in particular to explain the phenomenon of DNA condensation by polyamines [24]–[26]. Concerning microtubules, their self-attraction can also be triggered by multivalent salts and can lead to the formation of bundles [27]. The energy benefit of association between two like-charged bodies is generally due to the correlations between the multivalent counterions condensed on their surfaces. Here we will show that the interaction between two tubulin heterodimers, which are highly negatively charged proteins with a net charge about 20–30 e^−^, at pH around 7 [19], may also be influenced by this mechanism. Let us first estimate the attraction energy between two tubulin heterodimers in the presence of polyamines. The αβ-tubulin heterodimer is a nonspherical globule, having dimensions of 46×80×65 Å with two long C-terminal tails (*∼35 Å*) [3]. Most of the tubulin charge, at least 40%, is concentrated in the C-terminal tails so that the charge distribution on its surface is non homogeneous. We then need to investigate the electrostatic properties of the C-terminal tails and of the remaining of the heterodimer molecule separately. As described in details in Text S1, we obtained after theoretical developments the energy gain for these two interactions. The attraction energy for two heterodimers without the C-terminal tails is positive for putrescine and spermidine and negative only for spermine (*−0.26 K_B_T*), which means a weak energy benefit. As this result was obtained by considering that there is only spermine on heterodimer surfaces, we might expect that the resulting attraction force is negligible in physiological conditions where spermine competes with other cations or proteins for tubulin neutralization. On the other hand, the attraction energy becomes significantly larger between two interacting tubulin C-terminal tails. We obtained in this case *U_C_* values of +4.7, −2.6, −5 and −6.2 *K_B_T* for cation valence *Z* = 1, 2, 3 and 4 respectively, showing the presence of an attraction energy when Z>1 and which increases with the valence of ions. The energy benefit per counterion in correlations remains lower than *K_B_T* for divalent putrescine (see Text S1) so that it could hardly induce an attraction force between C-terminal tails due to thermal agitation. On the other hand, we expect that trivalent spermine and tetravalent spermidine can induce a significant attraction force between the C-terminal tails of tubulin dimers (see Text S1 and Figure 1A).

As the correlations between monovalent cations cannot contribute to the attraction force, the replacement of multivalent cations at high monovalent salt concentrations by monovalent ones lowers the attraction force. The fractional surface concentration of the multivalent cations, which is the ratio of the multivalent counterion surface density to the total surface density of the counterions, is very sensitive to the counterion valence [28],[29]. Higher valence cations are generally better competitors for the surface neutralization than lower valence ones (see Text S2 and [28]). In addition, the monovalent salt concentration above which multivalent cations are removed from the surface increases with the surface charge density [28]. For the highly negatively charged C-terminal tails, assuming σ∼1 e^−^/nm^2^ and using the model of Rouzina and Bloomfield [28] (see Text S2, supplementary file), about 50% of the spermidine counterions are removed with 300 µM spermidine and 150 mM KCl.

#### Experiments: AFM imaging

**Figure 2:**
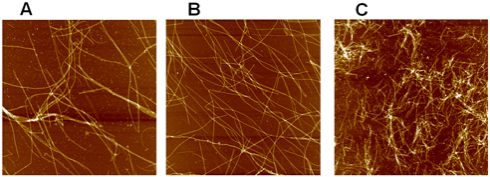
AFM images of microtubules assembled in the presence of various concentrations of spermidine in buffer M with 30 µM tubulin. Scan area: 8×8 µm^2^. (A) Control. (B) 200 µM spermidine. (C) 800 µM spermidine. Microtubules formed in the presence of moderate concentrations of spermidine (<*400 µm*) had a normal appearance without any tendency for bundling. Higher spermidine concentrations lead to the apparition of shorter MTs and some aberrant structures (*oligomers, aggregates, see also* Figure 3).

**Figure 3:**
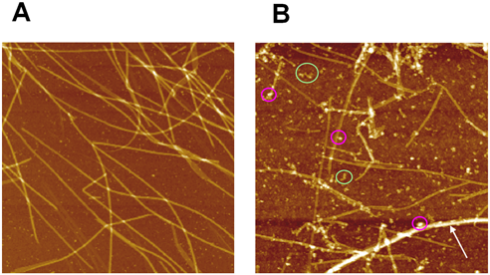
High resolution AFM images of microtubules in the presence of spermidine in buffer M. Scan area: 4×4 µm^2^. (A) 200 µM spermidine. (B) 800 µM spermidine. At moderate spermidine concentrations (*<400 µM*), long microtubules coexist with free tubulin dimers like in the absence of polyamines. Increasing polyamine concentration leads to the appearance of small tubulin oligomers or aggregates (*green circles*), microtubule bundles (*arrow*) and smaller microtubules. Interestingly oligomers or aggregates can also be adsorbed on MT surface (*pink circles*), as predicted for strong attractions (*Regime III*).

We first checked by atomic force microscopy if the presence of polyamines, in the physiological range, does not lead to aberrant microtubule structures. At low and moderate spermidine concentrations (<*400 µM in 25 mM MES-KOH pH 6.8, 20% glycerol*), we observed the formation of long microtubules like in the absence of polyamines (Figures 2A, 2B, and 3A). At higher concentrations of spermidine (>*400 µM*), very short microtubules, oligomers and (or) aggregates, and to a less extend MT bundles coexist (Figures 2C and 3B). It has already been observed that multivalent cations at high concentrations can promote the formation of large bundles [27]. The formation of small oligomers and (or) aggregates was also expected for a strong attraction regime (*Regime III, see “elongation” section*). The critical polyamine concentrations above which aberrant structures appear increase with the ionic strength. For example, in the presence of 800 µM spermidine and without KCl, small aggregates or oligomers are observed whereas, with 100 mM KCl, only long microtubules are observed at 800 µM spermidine.

### Polyamines Promote Microtubule Assembly via Electrostatic Interaction with the C-Terminal Tail

#### Effect of putrescine, spermidine and spermine on microtubule assembly

**Figure 4:**
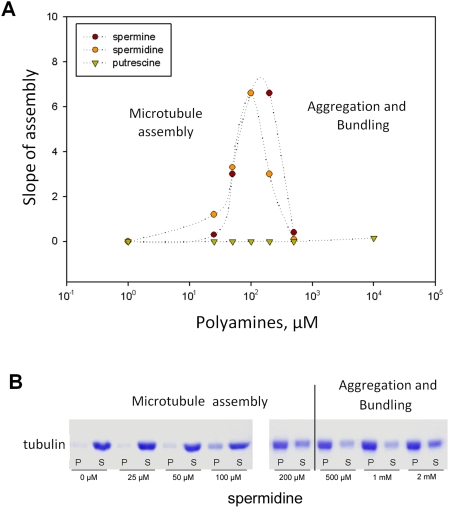
Effect of polyamines on the maximum slope of microtubule assembly. The tubulin concentration *(12 µM)* was purposely fixed to a value near the critical concentration in buffer M. (A) Both spermidine and spermine promote microtubule assembly at submilimolar concentrations whereas putrescine is unable to improve tubulin polymerization even at large concentrations (*>1 mM*). However, at high concentrations of spermidine and spermine (*>200 µM*), tubulin aggregates are formed which lowers the apparent assembly slope leading to a bell-shape profile. (B) SDS page analyses reveal that spermidine gradually increases the pellet mass. As observed by AFM, the increase of the pellet mass is due to microtubule formation at moderate spermidine concentrations (*25–200 µM*) but, at higher spermidine concentrations (*0.5–2 mM*), oligomers and (or) tubulin aggregates are also observed and participate to the increase of the pellet mass. The dashed line represents the transition between the zones of microtubule assembly and tubulin aggregation.

We measured the effects of polyamines on the maximum rate of microtubule assembly by analyzing light scattering curves and the mass of polymerized tubulin by sedimentation (*more specific aspects of microtubule assembly, notably nucleation and elongation, will be studied in the following sections*). It appears that spermidine (*Z = 3*) and spermine (*Z = 4*) promote MT assembly very efficiently while putrescine (*Z = 2*) has no effect up to 10 mM (Figure 4A). As previously reported, increasing the charge of the counterion favors MT assembly [17]. In agreement with our model, the effect of putrescine is negligible due to a lower energy gain in counterion sharing and an easier replacement by monovalent cations whereas the effects of spermidine and spermine on MT assembly are more pronounced. Above 150–300 µM, the beneficial effects of spermine and spermidine on the maximum polymerization rate decrease while they still induce a high polymerized mass (Figure 4B). However, under these conditions, we detected by AFM the presence of tubulin aggregates or oligomers in agreement with a non-zero value of the light scattering curve at the beginning of assembly (*data not shown*). Such a strong attraction between tubulin heterodimers is not due to a simple neutralization effect since neutralization does not lead to attraction between heterodimers. It should be triggered by an attraction force via polyamine correlations between tubulin dimers.

#### Influence of KCl on tubulin assembly in the presence of polyamines

**Figure 5:**
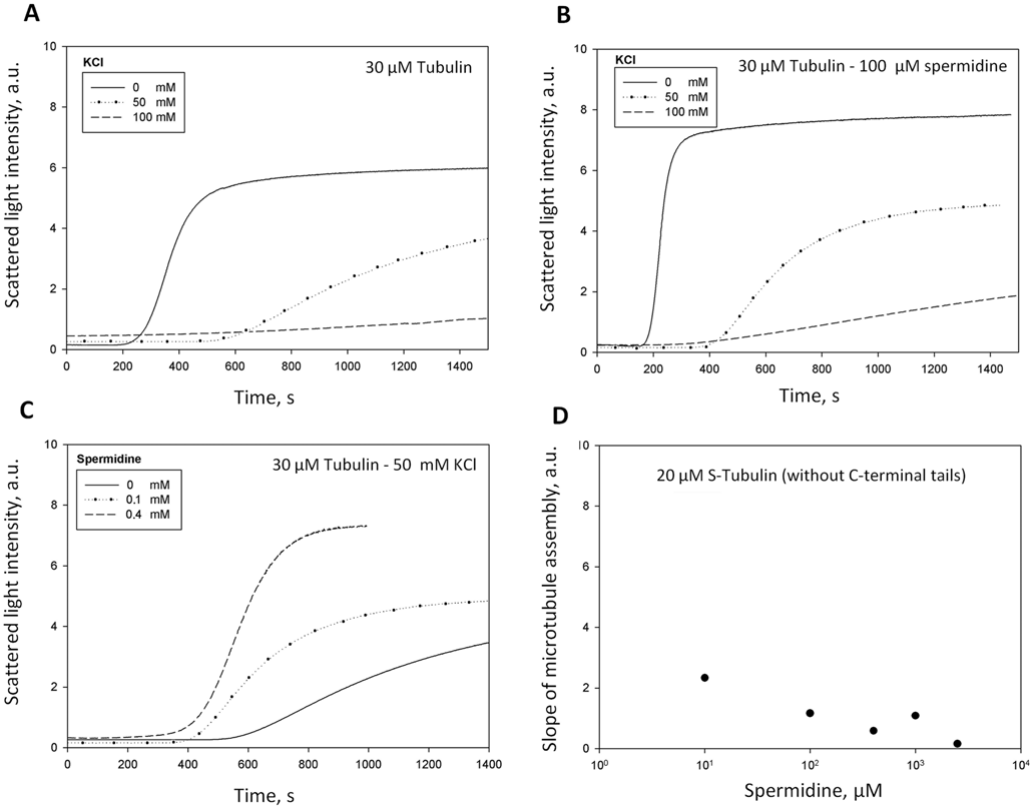
Effect of the competition between monovalent cations and multivalent polyamines on tubulin polymerization (*50 mM MES-KOH pH 6.8, 1 mM EGTA, 30 µM tubulin, 2 mM MgCl_2_, 20% glycerol*). (A) Inhibition of the tubulin assembly with the increase of KCl concentration. (B) Effect of KCl on tubulin assembly in the presence of 100 µM spermidine. The beneficial effect of spermidine is inhibited for KCl concentrations larger than 50 mM. We attribute this phenomenon to the replacement of polyamines by monovalent cations on tubulin surface which then inhibits the attraction between tubulin dimers (see Text S2). (C) To keep a high assembly rate at high ionic strengths, polyamine concentrations can be increased so that polyamine surface density on tubulin surface remains nearly constant. (AFM control revealed the presence of only microtubules under such conditions.) (D) Influence of spermidine on S-tubulin assembly. In the absence of the C-terminal tail, the beneficial effect of polyamine on tubulin assembly disappears.

Counterion correlations are known to be sensitive to the replacement of multivalent cations by monovalent ones. Therefore, upon increasing KCl concentration, the beneficial effect of polyamines on tubulin assembly should be significantly inhibited (see Text S2 for details). We thus analyzed the light scattering curves for different concentrations of KCl with or without spermidine. For all conditions, we verified using AFM that only MTs and not tubulin aggregates were formed. As predicted by our model, in the absence of KCl, the rate of assembly is higher with 100 µM spermidine than in its absence (compare Figure 5B with Figure 5A). In the presence of 50 mM KCl and 100 µM spermidine, the slopes of the assembly curves drop abruptly and the beneficial effect of spermidine is no longer observed. This is due to the replacement of polyamines by potassium ions which inhibits the polyamine sharing between tubulin dimers (see Text S2). To counteract the negative effect of KCl on tubulin assembly, the polyamine concentration can be increased (Figure 5C).

#### Involvement of the C-terminal tail of tubulin dimers

Another point which needs to be verified is the implication of the C-terminal tails of tubulin in the mechanism of counterion sharing between two tubulin heterodimers. We thus cleaved the charged C-terminal tails of tubulin by subtilisin [30] and observed that this led to the loss of the beneficial effect of spermidine on tubulin polymerization (Figure 5D). This indicates that the C-terminal tails are predominantly implicated in the mechanism of counterion sharing, which confirms previous results obtained with oligocations [17].

### Attraction of Tubulin Dimers onto Microtubules Mediated by Polyamines

**Figure 6:**
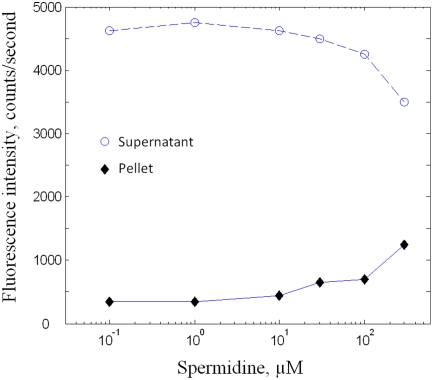
Attraction of tubulin dimers onto microtubule mediated by spermidine. 0.3 µM of Cy3-labeled tubulin was allowed to interact with 4 µM of non fluorescent taxol-stabilized microtubules at various concentrations of spermidine (*buffer M without GTP and without free taxol*). The fluorescence intensity of both pellets and supernatants were then measured after centrifugation at 20,000×g for 10 min. The results show that Cy3-tubulin co-precipitated with microtubules at spermidine concentrations higher than 10 µM (*under the conditions tested, we measured that no fluorescent tubulins sedimented in the absence of microtubules*).

As facilitated diffusion requires tubulin sliding on microtubules, we investigated if polyamines can mediate an attraction of tubulin dimers on microtubules. To measure this interaction, fluorescent Cy3-labeled tubulin (*0.3 µM*) was allowed to interact with non fluorescent taxol-stabilized microtubules (*4 µM polymerized tubulin*). This step was conducted without GTP at 22°C and without taxol to avoid the formation of new microtubules with Cy3-tubulin dimers. We then detected the attraction of fluorescent tubulin through the co-precipitation with microtubules at 20,000×g for 10 min at 22°C. The analysis of the fluorescence intensities of both supernatants and pellets shows than Cy3-tubulin was indeed co-precipitated with MTs at spermidine concentrations higher than 10 µM (Figure 6). Even if the supernatant fluorescence intensity is lower at higher spermidine concentrations, we note that the concentration of free tubulin in the supernatant remains close to its maximum at low spermidine concentrations. Therefore the attraction of tubulin onto microtubule is relatively weak under these conditions, with both free and adsorbed Cy3-tubulin coexisting. We shall see in the “elongation” section that the strength of this interaction is an important parameter which modulates the efficiency of facilitated diffusion. As a control, figure S1 in supplementary files shows more directly the attraction of Cy3-tubulin on a MT pellet mediated by 200 µM spermidine.

### Facilitated Nucleation

#### Introduction

In contrast to elongation, which can be considered as a pseudo-first order reaction process, the nucleation step is a more complex mechanism which requires the association of a few tubulin heterodimers to form a stable nucleus. Based on previous models of actin polymerization [31],[32], Voter and Erikson developed a widely used model of two-dimensional nucleation [33]. They showed that the tenth time of the assembly curve (*the time necessary to reach 10% of the plateau value*) is expected to decrease at the n^th^ power of the tubulin concentration where, for their model, 2n−1 is the number of tubulin dimers in the critical nucleus. More recently, using a different approach, Flyvbjerg and co-workers considered nucleation as process with successive intermediate steps of assembly during which only free tubulins are added to the growing nucleus [34]. In this model, there is still a similar power law dependence but between the final mass of polymerized tubulin and the tenth time of assembly. In addition they proposed that the size of the stable nucleus can be derived from the initial slope of the assembly curve in a log-log plot, this slope being proportional to the number of successive steps required to form the stable nucleus [34].

Whereas it is accepted that the tenth time of assembly decreases with a power law dependence in tubulin concentration (*or the final mass of polymerized tubulin*), the size of the stable nucleus for *in vitro* assembly is still under debate [35]. A larger nucleus of 15 dimers was found in [34] contrary to others which found a smaller nucleus of about 6 dimers, see for example [36]. Recently, it was suggested that the nucleus is not a clearly defined structure and that the number of dimers forming the nucleus is rather an average value between many alternative pathways leading to microtubule elongation [37]. This important issue needs however to be tackled specifically. Here we will mainly focus our attention on the influence of facilitated diffusion on nucleation using both the models of Flyvbjerg and co-workers and of Voter and Erikson.

#### Influence of facilitated diffusion

Nucleation requires the association of a few dimers *and* implies that the encounters take place via 3D diffusion. The probability of formation of such improbable complex can however be significantly enhanced by facilitated diffusion [8]. Facilitated diffusion, as already demonstrated for oppositely charged proteins [6], requires an attractive interaction between the encounter pair (*nucleus and tubulin*) to be fully operational. It leads to an increase of the interaction lifetime after collision so that there is enough time for diffusion and rotation until the target site is found according to the geometrical constraint (see Figure 1B), assuming that the attractive interaction is weak and will not dramatically affect protein mobility. Hydrophobic and electrostatic attractions between tubulin dimers can play a similar role. For example the tubulin critical concentration is reduced in the presence of glycerol [38], an effect attributed to a lower water activity and enhanced hydrophobic attraction. Regarding electrostatic interactions, the high negative charge of tubulin is responsive for an electrostatic repulsion which is unfavorable for nucleation. Transforming electrostatic repulsion into electrostatic attraction using polyamines would surely increase the lifetime of interaction [39] between a new arriving GTP-tubulin and the nascent nucleus and thus increases the nucleation rate. An increase of the tubulin:nucleus interaction lifetime can be integrated in a simple approach. If the time lapse between two consecutive encounters is shorter than the lifetime of the tubulin:nucleus interaction per encounter, the association constant should weakly depend on the free GTP-tubulin concentration because there will be enough GTP-tubulin to supply specific sites. This occurs above a concentration designated here as C_L_, below which the association rate decreases more abruptly with the free tubulin concentration. This latter feature is typical of the facilitated diffusion and differs from the previous nucleation theories.

#### Experiments

**Figure 7:**
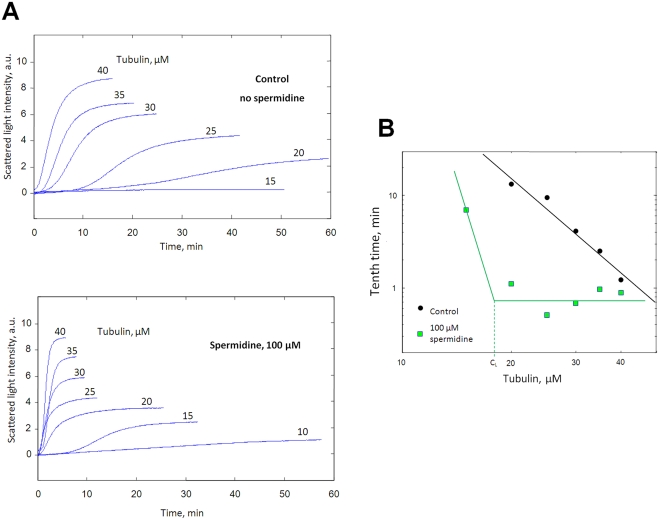
Facilitated nucleation mediated by polyamines. (A) Microtubules were assembled at various concentrations of tubulin with or without 100 µM spermidine at 37°C. (*25 mM MES-KOH pH 6.8, 1 mM EGTA, 30 µM tubulin, 2 mM MgCl_2_, 20% glycerol*). The samples were placed in a pre-warmed cuvette to initiate polymerization. (B) Log-log plot of tenth time versus tubulin concentrations extracted from (A). The experimental data are properly fitted by a straight line with a slope of −2.9 in the absence of spermidine. In contrast, in the presence of spermidine, the tenth time is nearly constant over a large range of tubulin concentration (*20–40 µM*), as represented by the horizontal line. Indeed the shortest nucleation duration is already reached at tubulin concentration larger than *C_L_* (∼*20 µM*) because of facilitated nucleation. For lower concentrations than *C_L_*, the tenth time increases sharply when the tubulin concentration decreases, as represented by the linear increase in the log–log plot.

Figure 7A shows the assembly curve for various concentrations of tubulin with or without spermidine. The measured value of the tenth time is represented in Figure 7B. The results are pretty different in control and in the presence of polyamine. Without spermidine, the log–log plot of the tenth time versus tubulin concentrations appears as a straight line in agreement with theories. In the presence of 100 µM spermidine, the tenth time is shorter than in the absence of spermidine whatever the tubulin concentration, thus suggesting that the rate of nucleation is globally higher. We also note that the tenth time decreases to a plateau value of about 0.5–1 min at tubulin concentrations larger than *C_L_* (*∼20 µM*). An increase of tubulin concentration has then little effect on nucleation duration, which indicates that 3D diffusion of tubulin to a nascent nucleus is no more the rate limiting step. As predicted by the model of facilitated diffusion, the flux of tubulin delivered to the nucleus may then be sufficient to sustain the highest rate of nucleation via facilitated nucleation. When the tubulin concentration decreases below *C_L_*, this is no longer the case since the tenth time increases.

**Figure 8:**
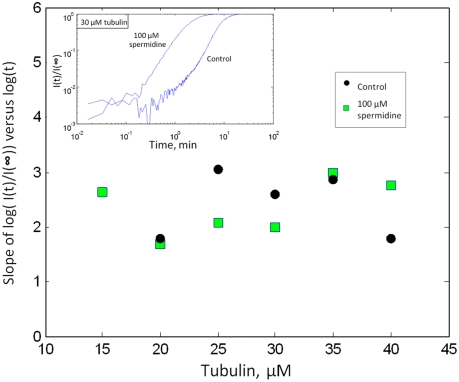
Scaling properties and nucleus size in the presence of spermidine. Initial slope of *log [(I(t)/I(∞)]* versus tubulin concentration. This slope is an interesting indicator of the number of tubulin dimers in the critical nuclei. The mean slope over tubulin concentrations is similar with or without spermidine, 2.36 and 2.32 respectively. Spermidine may then not affect the critical size of the nucleus but this remains to be demonstrated with a valid theory. Inset: examples of *log–log* plot of *[(I(t)/I(∞)].* The slopes were extracted at early times when the curves display a straight line.

As multivalent polyamines significantly influence the nucleation duration, we studied whether spermidine affects the critical size of the nucleus. In Figure 7B, the slope of the curve without spermidine, *n*, is about −3, which indicates that 5 dimers form the critical nucleus, in agreement with previous reports (the slope is about −2.4 in [35], −3.7 in [33] and −4 in [36]). However this curve could not be used to analyze the evolution of the critical nucleus in the presence of spermidine because the experimental data are not properly fitted by a straight line. To proceed differently, we use the theory developed by Flyvbjerg et al. [34]. Assuming some scaling properties of the assembly curves, Flyvbjerg et al. obtained that the critical size of the nucleus is then *3p*, where *p* is the slope of *log [I(t)/I(∞)]* versus *log(t)* (*(I(t)) is the scattered light intensity at a time t*). One of these scaling properties states that the tenth time scales like *I(∞)^−3^* (*equ. 3* *in ref* [34]), which indicates that nucleation duration is related to the final mass of microtubules. Our experiments confirmed this scaling property without spermidine but, in the presence of 100 µM spermidine, the power law dependence does not fit properly the data (see Figure S2). Facilitated nucleation explains this finding since the tenth time is roughly constant for tubulin concentrations higher than *C_L_*. In spite of this, we show in Figure 8 the slope of *[I(t)/I(∞)]* versus *log(t)* extracted from light scattering curves because it is an interesting indicator of the critical size of the nucleus [34],[35]. The results show that spermidine does not change significantly the mean value of the assembly slope at early times (Figure 8), 2.36 for control and 2.32 with spermidine. This leads to a critical nucleus of about 7 dimers, slightly larger than 5 dimers obtained in Figure 7B. The critical size of the nucleus may then be not affected by the addition of spermidine. However, in the absence of a valid theory, this prediction remains to be tested.

In summary, the important results of this section are: (i) nucleation rates are higher in the presence of spermidine compared to control, (ii) with spermidine, the power law dependence between the tenth time and tubulin concentration is no longer valid at tubulin concentrations higher than *C_L_*, contrary to previous nucleation models but in agreement with a facilitated diffusion mechanism.

### Facilitated Diffusion to the MT Ends

The probability that free GTP-tubulin molecules encounter long microtubule cylinders can be significantly larger than that of a direct encounter with MT ends. Therefore, after the encounters of tubulin with the MT wall, if the GTP-tubulin can proceed to the random scan of the MT via surface diffusion, a higher association rate of the free GTP-tubulin to the MT ends is expected (Figure 1C). A parallel can be drawn here between DNA and MTs. Indeed it has been shown that facilitated diffusion of site-specific proteins via 3D diffusion and 1D diffusion on long DNA increases their association rate to target site up to two orders of magnitude [40]. In recent theoretical studies [40],[41], simple equations have been developed to describe qualitatively the effect of facilitated diffusion on DNA/protein association constant, in particular, the useful model developed by Hu et al. [42] which describes in a simple manner the influence of the attraction energy on facilitated diffusion. Via straightforward modification of this model, we extend its domain of application to free GTP-tubulin diffusion to the MT ends. As the persistence length of MTs is larger than 1 mm [43], the MT is considered as a rigid cylinder. In addition, contrary to DNA, its larger radius implies that facilitated diffusion proceeds through 2D diffusion on the MT wall. The main assumption of this model is to consider that the flux of proteins delivered by 3D diffusion to the MT extremities at a distance lower than *λ* from one end is equal to the flux of protein delivered to the end via surface sliding at the equilibrium [42]. Considering *λ* as the sliding distance on the MT wall, if the separation distance from the nearest MT end and the new coming GTP-tubulin is shorter than *λ*, the GTP-tubulin can find its target site through 2D diffusion whereas for larger distances it will be released from the MT wall before reaching it. Then:
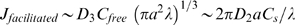
(1)where *D_3_, D_2_* are the tubulin diffusion constants in the bulk solution and at the MT surface respectively, *λ* is the *2D* diffusion length, *a* is the microtubule radius, *C_free_* is the free tubulin concentration, *C_s_* is the surface density of tubulin adsorbed on the MT surface. The first term of this equation is the *3D* diffusion near the MT extremities, and the second term represents the sliding.

Three different regimes can then be derived (see Text S3):

#### Regime I: *L*≪*λ*

**Figure 9:**
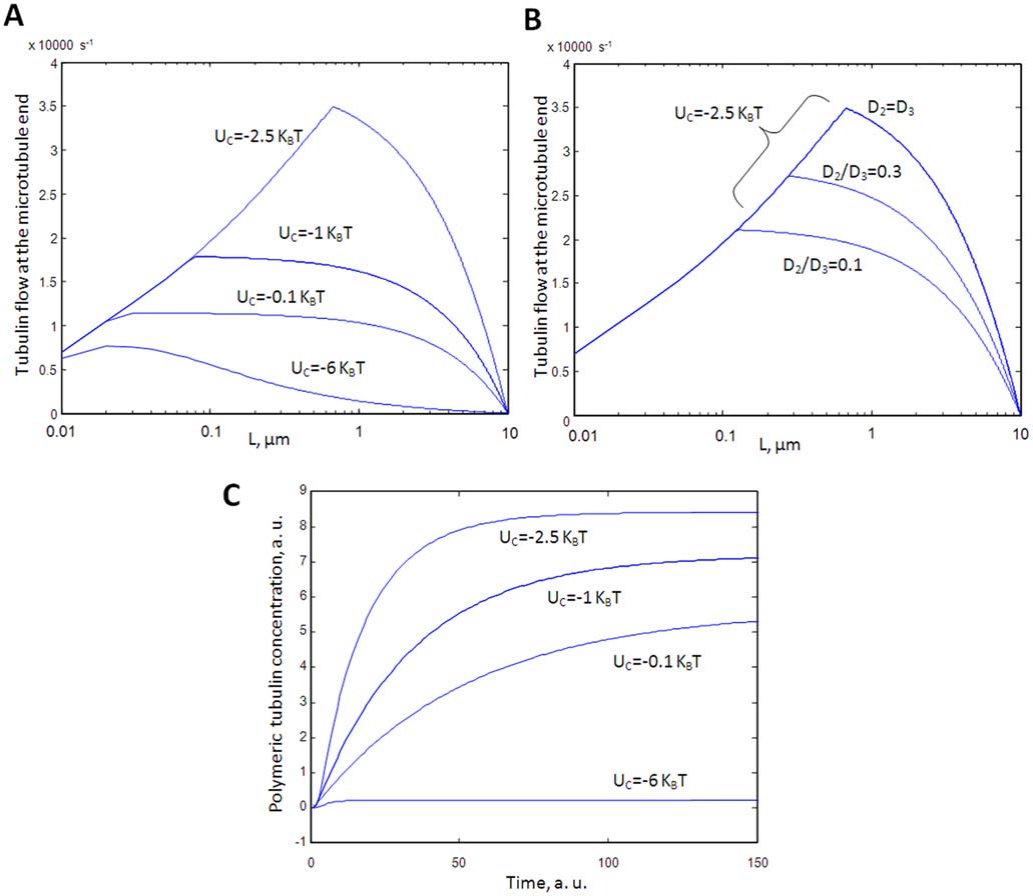
Effects of facilitated diffusion on elongation. (A) Facilitated diffusion of free GTP-tubulin to the MT ends versus mean MT lengths for different absorption energies (*U_C_*). For *Uc = −0.1 K_B_T*, the effect of facilitated diffusion can be neglected. For *Uc = −1 and −2.5 K_B_T*, diffusion of tubulin to the MT ends is facilitated by sliding. When the MTs are short i.e. at the early stage of MT polymerization, the regime I prevails, which is characterized by an increase of *J_facilitated_* with MT length. For longer MTs, for example *L>0.1 µm* for *Uc = −1 K_B_T*, the facilitated diffusion is not increasing with L anymore (*Regime II*). The sharp decrease of *J_facilitated_* when MT length approaches its maximum length (*10 µm*) is due to the low free GTP-tubulin concentration near the plateau of the assembly curve. The regime III occurs for *Uc = −6 K_B_T* and we can observe a rapid decrease of *J_facilitated_* with L. It is worth noting that we plotted the average values of the facilitated diffusion to the MT ends versus the average length of the MTs. In other words, it should not be confused with the elongation rate of individual MT. Parameters: *L_maximun_ = 10 µm, [tubulin] = 15 µM, D_3_ = D_2_ = 5.10^−12^* *m^2^s^−1^, e = 4 nm*. (B) In (A), we assumed that D_3_ = D_2_. The benefit of facilitated diffusion was then maximum. If we now consider that D_2_/D_3_ = 0.3 or D_2_/D_3_ = 0.1 due to hindered diffusion on microtubules, we observe that the transition from regime I to regime II will arise at shorter *L values.* This partly inhibits the beneficial effect of facilitated diffusion of the elongation rate. *D_3_ = *5.10^−12^ m^2^s^−1^. (C) Simple model of microtubule assembly versus incubation time for three attraction energies, which points out the influence of facilitated diffusion on the MT elongation. For this purpose, it is arbitrary assumed that the mean number of MT nuclei is the same for the three different conditions (see Text S3). It can be though as the last part of the light scattering curve, after the inflexion point, which is more elongation-sensitive. In addition, we assume that the elongation rate is proportional to the difference (*J_facilitated_ -J_0_*), where *J_0_* is the critical flux of GTP tubulin for which the elongation rate equals the shortening rate. Indeed it is has been shown both experimentally and theoretically that increasing the GTP-tubulin concentration above the critical concentration leads to a linear increase of the mean elongation rate [45]. This figure shows that facilitated elongation of MTs through GTP-tubulin sliding both results in a higher amount of polymerized tubulins and a more abrupt slope near the plateau value. Parameters: *J_0_ = 5000 s^−1^; e = 4 nm; [tubulin] = 15 µM; D_3_ = D_2_ = 5.10^−12^* *m^2^s^−1^; a = 12 nm.*

This regime occurs at the early stage of MT assembly when the mean MT length (*L*) is very short (*L<λ∼100 nm for U_C_≈−1.2 K_B_T using equ. C2 and C3 in* *Text S3* *with e = 4 nm, a = 12 nm, D_3_ = D_2_*), which corresponds to the elongation step just after nucleation. Provided that MT is shorter than the diffusion length *λ,* every time a free tubulin touches the MT surface via 3D diffusion, it is able to find its binding sites at the MT extremities via sliding:
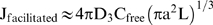
(2)The diffusion to the MT ends does not apparently depend on the attraction energy but the influence of *U_C_* is to set the critical length *λ* (*see equ.C2 and C3 in* Text S3) up to which we quit the Regime I. As *λ is* longer for stronger attraction, it indicates that regime I is valid for longer MTs under such conditions. It thus results in a highly beneficial effect of facilitated diffusion since the diffusion to the MT ends increases proportionally to *L* (see Figure 9A). We also note that *λ* scales like *D_2_^3/4^* (see *equ.C2* Text S3). A decrease of *D_2_* due to surface friction (*strong attraction*) on MT surface could then restrict the domain of validity of Regime I to shorter microtubules. Consequently, facilitated elongation loses part of its effectiveness (see Figure 9B). Let us remark that *D_2_* is a difficult parameter to estimate theoretically and to measure experimentally.

#### Regime II, *L*≫*λ* and moderate attractions (2πaLey<v, with 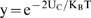), see Text S3 for details

When MTs are longer than λ and for moderate attractions, we enter in the second regime. For example, regime II takes place when U_c_>−4 K_B_T for L = 5 µm and v = 2 µm^3^ (volume per microtubule). Under such conditions:
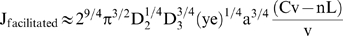
(3)where *C* is the total tubulin concentration, and *n* is number of tubulin dimers per *µm* of microtubule. In this regime the facilitated diffusion leads to an increase of the diffusion rate to the MT extremities with *y^1/4^,* 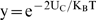. The point is that the facilitated diffusion depends weakly on *L* and depends linearly on free tubulin concentration. However, when *nL∼C*v, the free tubulin concentration is very low and the association rate drops rapidly with the mean MT length so that the mean MT length reaches a plateau value (see Figure 9A).

#### Regime III, *L*≫*λ* and strong attraction (2πaLey>v), see Text S3 for details

In this case, there is a sequestration effect of increasing the number of adsorbed tubulin which reduce the pool of free GTP-tubulin concentration and slow down association to the MT ends.
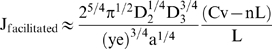
(4)The diffusion rate is now decreasing with the MT length and with the attraction force (*y*).

This model neglects the presence of oligomeric structures. Small oligomers reduce the pool of free tubulin dimers and their diffusion is slower than that of tubulin dimers, which slows the elongation rate. At low magnesium concentrations, the effect of oligomerization is of little importance, as recently shown [44] in the presence of 1 mM MgCl_2_, but, at high polyamine concentrations (*regime III*), the formation of tubulin aggregates or small oligomers may be favored (see Figure 3B). In the regime III, in addition to the sequestration effect, the formation of multimeric structures could thus lower the elongation rate of microtubules.

To summarize the influence of facilitated diffusion on elongation, Figure 9A represents the rate of GTP-tubulin arriving to the MT ends via facilitated diffusion versus the microtubule length for four attraction energies, (*U_c_ = −0.1 K_B_T, very low; U_c_ = −1 K_B_T, low; U_c_ = −2.5 K_B_T, moderate; U_c_ = −6 K_B_T, very strong*). Facilitated diffusion can increase significantly the flux of protein arriving to the MT ends for low and moderate attractions (*at L∼1 µm, there is roughly a fourfold increase of J_facilitated_ between Uc = −0.1 K_B_T and −2.5 K_B_T)* but is not beneficial for strong attractions (*Uc = −6 K_B_T)*. The flux of GTP-tubulin can be related to the MT elongation rate by assuming that the supply of GTP-tubulin is the rate limiting step for MT elongation. It is certainly a rough assumption, though in agreement with experiments showing an increase of the elongation with GTP-tubulin concentration [45], but it is useful to decipher the possible influence of facilitated diffusion on elongation. For example we expect that a strong attraction should induce shorter MT since the flux of GTP-tubulin is significantly smaller for longer MT in the regime III. We can even go a little further and plot the evolution of the MT assembly curve versus time which only arbitrary takes into account a differential elongation. To do so, we consider that stable nucleus are already formed at *t = 0* and are at the same concentration whatever the polyamine concentrations (see Text S4). These hypotheses allow us to observe the influence of elongation only. Figure 9C shows that facilitated elongation could potentially increase the plateau value and the steepness of the assembly curve.

In our simple analytical treatment, we neglect the high electric dipole moment of tubulin heterodimers. Microtubules are indeed asymmetric and the local electric field generated by tubulin dipoles should influence the orientation of interacting dimers. Microtubule polarity is also known to cause differential elongation rates between the plus (*fast growing end*) and the minus ends (*slow growing end*). As facilitated elongation proceeds via tubulin sliding on microtubules, microtubule polarity may bias the random sliding with a preferential direction. The relation between facilitated diffusion and local electric fields on microtubule surface could then be an interesting subject of future investigations.

#### Experiments

**Figure 10:**
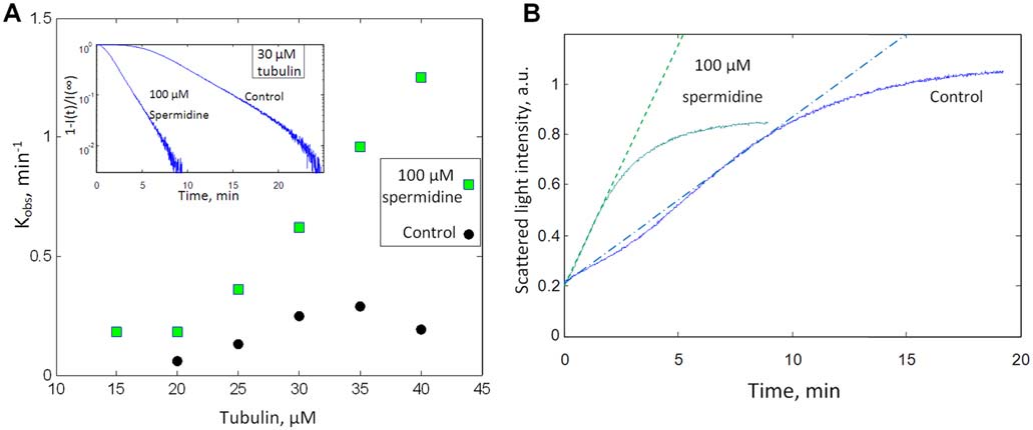
Effect of spermidine on microtubule elongation. (A) Pseudo-first order rate constant of elongation, *K_obs_*, versus tubulin concentration in buffer M. 100 µM spermidine significantly increases the elongation rates whatever tubulin concentration. Inset: *Log-plot* of *1−I(t)/I(∞)* versus time for 30 µM tubulin. The slope of this curve is −K_obs_. The elongations rate is about three times higher with spermidine (*except for 40 µM tubulin*). (B) A microtubule solution was prepared by incubating 30 µM tubulin at 37°c for 1h in 50 mM MES-KOH pH 6.8, 1 mM EGTA, 2 mM MgCl_2_, 20% glycerol, 1 mM GTP. At the end of the incubation, 50 µl of a solution containing 30 µM of free tubulin dimers without or with 100 µM spermidine was then added to 50 µl of the microtubule solution. The sudden increase of the free tubulin concentration allows one to observe elongation of the preformed microtubule via light scattering. It turns out that the presence of 100 µM spermidine leads to a significant increase of the elongation rate as indicated by the slope of the assembly curve, which is 2.7 times higher with 100 µM spermidine (*see dotted lines*). The plateau value which is more rapidly reached in the presence of polyamines also evidences a facilitated elongation.

According to the model of facilitated diffusion to the MT ends (Figure 9), the light scattering curves should reach their plateau value more abruptly in the presence of polyamines. The experiments presented in Figure 7A clearly show that the plateau value is reached quickly in the presence of polyamines. Therefore facilitated diffusion seems to have a beneficial effect on MT elongation for moderate attraction forces between tubulin dimers. However stronger evidences are required to confirm this proposal of facilitated elongation. We thus analyzed the pseudo-first order rate constant of elongation, *k_obs_*. This parameter can be obtained by measuring the slope of *log [1−(I(t)/I(∞)]* as a function of time [35]. Figure 10A shows that the pseudo-first order rates of elongation increase about 3 times in the presence of 100 µM spermidine whatever the tubulin concentration (*except for 40 µM, the elongation rate is then lower than expected without spermidine*). In addition, we note that the pseudo-first order rate constant of elongation increases with tubulin concentration in the presence of spermidine, as predicted in Regime II.

To more directly access to elongation and limit the influence of nucleation, we measured the elongation rate of preformed microtubules by adding free tubulin to a microtubule solution. Under such conditions, free tubulin will mostly participate in the elongation of preformed microtubules due to the sudden increase of the free tubulin concentration. If 100 µM spermidine is added to the microtubule solution simultaneously with free tubulin (see Figure 10B), the elongation rate is significantly increased compared to control. In addition, the plateau value is reached more rapidly thus indicating a rapid consumption of the free tubulin pool in agreement with the model of facilitated diffusion to the MT ends.

#### Implication for MT stabilization

**Figure 11:**
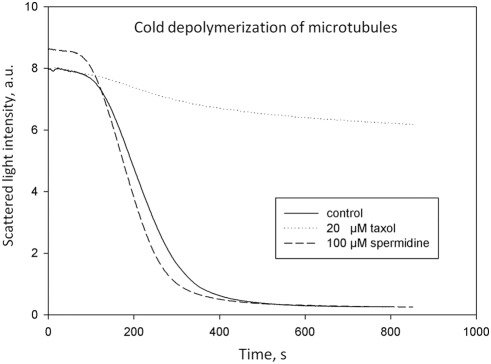
Microtubule instability in the presence of polyamines. To estimate the stability of microtubules, the time course of cold disassembly at 8°C of pre-assembled microtubules (*35 µM tubulin*) is reported in the presence or absence of 100 µM spermidine. The rate of disassembly is not affected by the presence of 100 µM polyamines whereas it is significantly reduced in the presence of 20 µM taxol, a well known stabilizing agent. These results are thus in agreement with our model since polyamines were not expected to significantly favor microtubule stability and thus should preserve microtubule dynamical instability.

An attractive interaction mediated by polyamines may increase the flux of new arriving GTP-tubulins to MT ends. The GTP cap should then be larger which in turn favors microtubule stabilization. However, beyond this kinetic effect, the MT structure would be more stable if an attraction force between tubulin dimers could prevent microtubule depolymerization. During depolymerization, the lateral bonds between dimers (−*3.2 to −5.7 K_B_T*) first dissociate because they are significantly weaker than longitudinal bonds *(−6.8 to −9.4 K_B_T*) [46]. To test if the electrostatic interactions mediated by polyamines can influence the depolymerization mechanism, we measured the rate of microtubule disassembly at low temperature with or without spermidine and found that spermidine does not significantly lower the rate of MT disassembly (see Figure 11). This suggests that polyamines do not behave like stabilizing agents. A probable explanation for this is that the roots of the C-terminal tails of two consecutive heterodimers in the MT wall are separated from each other by a distance larger than 5 nm in the lateral direction [47], which implies only very limited possibility for the overlapping of two C-terminal tails and thus for polyamine correlations to take place between two C-terminal tails. This observation is thus in agreement with our theoretical conclusion.

After incorporation in the MT wall, GTP-tubulin is hydrolyzed into its inactive form, GDP-tubulin. As the presence of GTP-tubulin at the MT ends, the “GTP cap”, is necessary to stabilize microtubules, a sufficient supply of GTP-tubulin to the ends of growing MTs needs to be maintained. In case of lack of GTP-tubulin supply, the GTP cap is then hydrolyzed into GDP-tubulin, which increases the probability of shortening. Microtubule shortening results in an important release of GDP-tubulin from microtubule wall and, in this context, facilitated diffusion can play an important role. For example, when a microtubule shrinks, the attraction on its surface of its own GDP-tubulins may lower the probability of rescue.

#### Microtubule assembly in the presence of MAPs and polyamines

**Figure 12:**
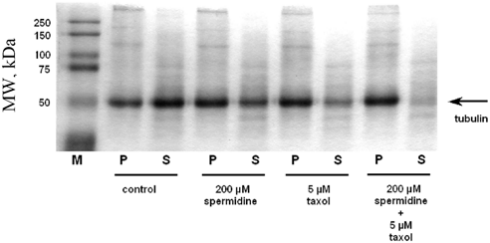
Influence of spermidine on MAPs-tubulin polymerization. MAPs-tubulin (*12 µM tubulin*) was assembled into microtubules as described in *materials and methods* and then the tubulin content was examined in the supernatant (*S*) and the pellet (*P*) after high speed centrifugations. A volume of cold PBS (*4°C*) equal to that of supernatant was added to pellet. 8 µL of each solution were loaded. The addition of 200 µM spermidine increases the amount of polymerized tubulin even in the presence of MAPs. Furthermore a mixture of taxol and spermidine leads to an additional amount of polymerized tubulin. (*M*) Molecular weight markers.

Microtubule associated protein regulates microtubule dynamics *in vivo* and may thus inhibit the effect of polyamines on tubulin polymerization. However, it was reported that polyamines can still enhance tubulin polymerization in the presence of MAPs [48]. To further document this report, we examined *in vitro* the effect of polyamines at physiological concentrations on MAPs-microtubules and it appears that they indeed enhance tubulin polymerization (Figure 12).

As polyamines facilitate nucleation and elongation and do not stabilize MTs, it is expected that the presence of a stabilizing agent (*taxol*), polyamines should also further improve tubulin assembly. We then observed the effect of spermidine on MAP-tubulin assembly in the presence of taxol. It turns out that a mixture of taxol and spermidine leads to a larger amount of polymerized tubulin than with taxol alone, in agreement with a previous report [48]. However MAPs are known to partly neutralize microtubules via their binding to the C-terminal tails of tubulin heterodimers. MAPS can therefore lower the attraction between tubulin dimers and microtubules mediated by polyamines. Further studies are also needed to decipher how the presence of MAPs may affect diffusion of free tubulin on microtubules.

## Discussion

To describe microtubule assembly and dynamics *in vitro*, a simple model with two phases, nucleation and elongation is generally advanced. Nucleation proceeds via the assembly of several tubulin dimers into a nucleus of critical size [33],[34]. The size of this nucleus varies with the ionic composition of the buffer and the presence of tubulin partners. Above this critical size, a short microtubule can be formed and then the elongation of the microtubule takes place via the addition of free GTP-tubulin to its extremities [33],[34],[36],[37], which leads to the formation of a long MTs. The elongation phase is nearly completed when the flow of new incoming GTP-tubulin is not sufficient to maintain the GTP cap at the MT ends [45],[49],[50] and thus to prevent MT depolymerisation [45].

The important point is that microtubule assembly requires the constant supply of GTP-tubulin dimers via 3D diffusion to nucleus and MT ends. The rate of encounters between tubulin dimers and a body of radius b_0_ (*nucleus or MT ends)* is given by the well known Smoluchowski equation: *J_s_ = 4πD_3_C_0_b_0_,* where *D_3_* is the 3D diffusion constant of tubulin and C_0_ is the concentration of free tubulin. Using *b_0_≈4–10 nm (4 nm is the value previously used to describe the tip size at the MT ends* [51] *)*, *D_3_≈5.10^−12^ m^2^/s* and *C_0_ = 10 µM* (*measured in sea urchin egg extracts* [7]), we obtain about 1500–3800 collisions per second. Such a high collision rate via 3D diffusion is largely enough to sustain typical elongation rates (about *14 µm/min* [10],[51]). Indeed, there are about 1640 subunits per µm of MT and, if we assume that both ends grow at the same rate, thus neglecting treadmilling, the association rate of free GTP-tubulin to one end needs to be only larger than 190 s^−1^ to maintain elongation [51]. It could then be advanced that MT dynamics is not diffusion limited. However, this conclusion is in apparent contradiction with several experimental evidences linking the GTP-tubulin supply to the MT ends and the mean rate of elongation. First, upon dilution, MTs collapse [52]. Second, near critical concentration, the GTP-tubulin supply becomes so critical that elongation only compensate shortening [45]. Third, the larger is the free tubulin concentration, the faster is the elongation [45] and the rate of tubulin nucleation also increases sharply with the free tubulin concentration [33],[36]. One possible explanation for the discrepancy between the calculated high collision rate and the high sensitivity of both nucleation and MT elongation on free tubulin concentration is that the probability for a new coming tubulin to dock correctly on its target site (*lateral or longitudinal bounds*) is low [9] and thus requires several collision trials. To add credit to this idea, theoretical and experimental arguments have shown that an encounter can evolve into a stable complex only if the two interacting biomolecules are correctly oriented and positioned, with respect to theirs binding sites [8]. This geometrical constraint, which cannot be neglected in the case of tubulin and microtubule, leads to an estimated probability of complex formation per encounter 3 to 6 orders of magnitudes lower for typical proteins, depending on the number of binding sites and their dimensions [6],[8]. Taking into account this geometrical factor, the net tubulin association rate to the nucleus or MT ends in the conditions described above would rather be between 0.0015–0.0038 and 1.5–3.8 s^−1^, which is not sufficient to sustain MT assembly (*190 s^−1^*). In this context, it is difficult to longer consider that MT assembly is not diffusion limited since many more encounters need to occur to allow microtubule elongation and the formation of critical nucleus. As an attraction between tubulin dimers can be mediated by multivalent counterions, we propose that polyamines could significantly increase the elongation and nucleation rates via facilitated diffusion mechanisms.

Facilitated diffusion increases the flow of free tubulin arriving at the MT ends through tubulin sliding. As we can reasonably assume that a larger flow of tubulin favors elongation, a significant increase of the elongation rate is then expected under the best conditions for facilitated diffusion (see Figure 9A). Such theoretical predictions are in close agreement with the experimental results presented in Figure 10. A threefold increase of the pseudo-first order rate constant of elongation was measured experimentally with 100 µM spermidine. Such an increase could be critical *in vivo* when tubulin supply is scarce. We also observed that the pseudo-first order rate constant of elongation constantly increases with tubulin concentration in the presence of spermidine. It indicates that microtubule ends are not saturated with free tubulin and, therefore, elongation is still diffusion-limited in the presence of polyamines. The elongation rate could then be further increased up to GTP-tubulin saturation, which may arise at very high concentrations of tubulin.

For the nucleation step, an attraction force mediated by polyamines induces a longer interaction lifetime between a new incoming GTP-tubulin and the forming nucleus. In agreement with this, experimental results clearly indicate that nucleation duration in the presence of polyamines is significantly decreased compared to control (Figure 7), whereas, the critical size of the nucleus may not be affected (Figure 8). Interestingly, the tenth time (*lag time of assembly*) no longer decreases for tubulin concentrations higher concentration than *C_L_* (*∼20 µM*, Figure 7B). This is an apparent discrepancy with classical theory and with typical *in vitro* experiments as a slight reduction of the pool of free tubulin increases the lag time by a dramatic factor (Figure 7 and [33],[34],[36]). In contrast to elongation, the formation of a stable nucleus is the result of many relatively stable intermediate aggregates [34]. During this slow process, free tubulin dimers could accumulate on growing nucleus in the presence of polyamines. Consequently, the rate of nucleation no longer depends on GTP-tubulin concentration because of over-supply. This results is in agreement with *in vivo* reports from yeast showing that these cells do not have very sensitive mechanisms to regulate their tubulin level (*after a two-fold reduction*), and that such regulation was not necessary for normal microtubule function [53]. The discrepancy between the necessity for a close regulation of free tubulin *in vitro* and the apparent independence of the nucleation rate on free tubulin level *in vivo* could thus be partly explained by polyamines beside other nucleation factors such as γ-tubulin [54]. The reciprocal consequence is that modulation of the polyamine level should influence microtubule nucleation.

Another interesting point raised by the present study is that polyamines do not significantly stabilize microtubules (Figure 11). Indeed, polyamines participate to MT dynamics to both enhance nucleation and the formation of long microtubules but, in the same time, they do not prevent MT collapse which would have been toxic for living cells. The absence of a significant stabilizing effect of polyamines on microtubules was at first glance surprising; however it is most probably due to the fact that, in the MT wall, the distance between the C-terminal tails of adjacent tubulin heterodimers is too long to allow their significant overlapping.

The model of facilitated diffusion provides basis to enlighten the interplay between polyamines and microtubule dynamics in living cells. The polyamine levels vary greatly during the cell cycle and in relation to the proliferation status of tissues [14],[15],[17],[55], which may significantly impact microtubule dynamics in living cells [15],[16],[56]. Indeed it has been shown that polyamine concentrations increase significantly during mitosis with a sequential patterns reflecting the fact that putrescine is a precursor of spermidine and spermidine in turn is a precursor of spermine [15]. In parallel to the increase of polyamines during mitosis, the rate of microtubule nucleation also increases sharply during mitosis [57] to reach at anaphase a sevenfold increase compared to that observed in the G2 phase. Our results suggest that the increase of the polyamine content could support the increase of MT nucleation and elongation. Against this proposal it could however be argued that MAPs, which are known to neutralize the negative charge of microtubules and to stabilize the microtubule wall, are such strong competitors for the microtubule neutralization that they might inhibit the overall effects of polyamines. However our results show that polyamines influence MT dynamics even in the presence of MAPs (Figure 12). Interestingly we also observed that the combination of taxol and spermidine cooperate to increase the microtubule mass, which indicates that polyamines and taxol could use different mechanisms (*facilitated diffusion and stabilization, respectively*).

In summary, our results lead to the following conclusions:

1. An attraction between two tubulin heterodimers is induced by the presence of multivalent counterions due to their sharing between the highly negatively charged C-terminal tails of tubulin. Under living cell conditions, this can take place in the presence of natural polyamines (*spermidine and spermine*).
2. A moderate attraction force generated by polyamines enhances the rate of GTP-tubulin arriving to the MT ends via facilitated diffusion and thus the rate of microtubule elongation. Indeed, GTP-tubulin first proceeds through 3D diffusion to find the MT surface and then slides on the MT wall to reach its extremities.
3. Due to an electrostatic attraction, the microtubule nucleation process is facilitated via an increase of the interaction lifetime between a new incoming tubulin and the growing nucleus thus increasing the probability of incorporation of tubulin in the nucleus. Above a critical concentration *C_L_*_,_ we observe that the nucleation duration no longer decreases with the tubulin concentration.
4. Polyamines seem to have little effect on MT stability which can be due to the large distance between the C-terminal tails of tubulin inside the MT wall.

## Materials and Methods

### Chemicals

Polyamines (*putrescine, spermidine, spermine*) were purchased from Sigma-Aldrich and used without further purification.

### Tubulin Preparation

Tubulin was purified from sheep brain crude extracts as described previously [58]. For long term storage, aliquots were stored at −80°C in 50 mM MES-KOH pH 6.8, 0.5 mM dithiothreitol, 0.5 mM EGTA, 0.25 mM MgCl_2_, 0.5 mM EDTA, 0.1 mM GTP, 30% glycerol (*v/v*). Before use, an additional cycle of polymerization was performed in 50 mM MES-KOH pH 6.8, 0.5 mM dithiothreitol, 0.5 mM EGTA, 6 mM MgCl_2_, 0.5 mM EDTA, 0.6 mM GTP, 30% glycerol. Microtubules were sedimented by centrifugation (*52,000×g, 30 min at 37°C*) at the end of which the microtubule pellet was resuspended in 25 mM MES-KOH pH 6.8, 0.5 mM EGTA, 1 mM DTT and disassembled at 4°C for 20 minutes. Tubulin aggregates were finally eliminated by a further centrifugation at 4°C (*52,000×g, 20 min*). Tubulin concentration was determined by spectrophotometry using an extinction coefficient of 1.2 mg^−1^×cm^2^ at 278 nm [59].

### Microtubule Assembly

Pure tubulin was pre-incubated on ice for 2 min in buffer M (*25 mM MES-KOH pH 6.8, 20% glycerol, 1 mM EGTA, 2 mM MgCl_2_, 0.5 mM GTP*) in the presence or absence of various concentrations of polyamines. For Figures 4 and 5, tubulin polymerization was initiated by placing the ice-cold cuvette (1 *cm light path*) at 37°C in a PTI QuantaMaster 2000-4 thermostated spectrofluorimeter. The kinetics of microtubule assembly was then immediately monitored by 90° light scattering at 370 nm. For Figures 7, 8, and 10, the tubulin samples were immediately placed in a pre-warmed cuvette (*time t = 0*) to capture the kinetics of nucleation and elongation. This eliminates the time necessary for heating the cuvette in the spectrofluorometer.

### Preparation of S-Tubulin

S-tubulin was prepared as previously described [60]. Briefly, subtilisin was added to tubulin with a 1∶200 subtilisin∶tubulin (*w/w ratio*). The mixture was then incubated for 40 min at 25°C. In these conditions, about 95 % of tubulin was correctly cleaved by subtilisin as determined by gel electrophoresis (*percentage of cleavage*) and MALDI-TOF mass spectrometry (mass of cleaved tubulin). Aliquots of S-tubulin were used fresh or kept frozen in 25 mM MES-KOH pH 6.8, 1 mM EGTA, 10% glycerol.

### Effects of Polyamines on Tubulin Assembly by Sedimentation Assay

12 µM of pure tubulin was pre-incubated on ice for 2 min in (*25 mM MES-KOH pH 6.8, 20% glycerol, 1 mM EGTA, 2 mM MgCl_2_, 0.5 mM GTP)* with various concentrations of KCl and spermidine (as indicated). Tubulin assembly was obtained after incubation at 37°C for 20 min. Microtubules were then pelleted at 25,000×g for 15 min at 37 °C and resuspended in 25 mM MES-KOH at 4°C in a volume equivalent to that of the supernatant. Equal volumes of supernatant and resuspended pellet were loaded on SDS-PAGE.

### Cosedimentation Assay To Detect Tubulin Dimer/Microtubule Interaction

To analyze the attraction of Cy3-tubulin on microtubules, Cy3-tubulin was prepared as described previously to obtain 0.5 Cy3 molecules per tubulin dimer [61]. Non fluorescent microtubules were preassembled in the presence taxol and then pelleted at low speed (20,000×g for 10 min). The pellet was washed several times to remove free taxol. After resuspension in buffer M without GTP, taxol-stabilized microtubules (*4 µM of polymerized tubulin*) were then allowed to interact with 0.3 µM of Cy3-tubulin at various concentrations of spermidine. 100 µl of the reaction solutions were centrifuged at 20,000×g for 10 min to pellet MTs and MT-adsorbed Cy3-tubulin. Pellets were resuspended in the starting volume. Fluorescence intensities of pellets and supernatants were then measured using a PTI QuantaMaster 2000-4 thermostated spectrofluorimeter (λ_excitation_ = 550 nm, λ_emission_ = 569 nm).

### Effects of Spermidine and Taxol on MAPs-Tubulin Assembly Determined by Sedimentation

1.4 mg/ml of twice cycled tubulin containing 15% of MAPs was incubated 5 min on ice in 25 mM MES-KOH pH 6.8, 5 mM MgSO_4_, 1 mM EGTA, 1 mM GTP, 0.7 mM ATP, 1 mM DTT with or without 200 µM spermidine or/and 5 µM taxol. The samples were then placed at 37°C for 15 min. Microtubules were then pelleted at 25,000×g for 15 min. Pellets and supernatants were then analyzed by SDS-PAGE.

### AFM Imaging

Microtubules samples from assembly reaction (*in buffer M*) were directly deposited on NiCl_2_ pretreated mica surfaces. Samples were deposited on the mica surface for a short time of about 30 s to prevent microtubules from collapsing on the surface as previously reported [62]. Finally, the surface was then dried with filter paper.

AFM imaging was performed in intermittent mode. We used silicon cantilevers AC160TS (*Olympus*) with resonance frequencies of about 300 kHz. All images were collected at a scan frequency of 1.5 Hz and a resolution of 512×512 pixels. A first or second order polynomial function was used to remove the background slope.

## Supporting Information

Figure S1 Attraction of fluorescent tubulin dimers onto microtubule mediated by spermidine. Fluorescence microscopy of a non fluorescent MT pellet in the presence of 0.1 µM Cy3-tubulin in buffer M without GTP. i) Microtubule pellet alone; ii) 0.1 µM of Cy3-tubulin was allowed to interact with the microtubule pellet for 15 min; iii) Addition of 200 µM spermidine (15 min); iv) Addition of 300 mM NaCl (30 min). Following the addition of spermidine, we observe the apparition of the microtubule pellet in the fluorescence image. The signal to background ratio due to Cy3-tubulin attraction in the pellet is about 13% under such condition, as shown in the line profile of fluorescent intensity. As expected for an electrostatic interaction, Cy3-tubulin was partly released from the microtubule pellet upon the addition of NaCl up to 300 mM.
(0.16 MB TIF)

Figure S2 Log-log plot of the tenth time versus plateau value of the assembly curve extracted from Figure 7A. In the absence of polyamines, a straight line properly fits the experimental data. Its slope is about −3 in agreement with the results of Flyvbjerg et al [34]. This scaling properties is however not valid in the presence of spermidine.
(0.02 MB TIF)

Text S1 Attraction between tubulin dimers
(0.05 MB DOC)

Text S2 Competition between cations
(0.03 MB DOC)

Text S3 Facilitated diffusion
(0.04 MB DOC)

Text S4 Polymeric tubulin concentration
(0.02 MB DOC)
