# Supplementary figures and images for: Polyamine Sharing between Tubulin Dimers Favours Microtubule Nucleation and Elongation via Facilitated Diffusion
Source: PLoS Comput Biol. 2009 Jan 2;5(1):e1000255. doi: 10.1371/journal.pcbi.1000255 (PMC2599886; doi:10.1371/journal.pcbi.1000255)

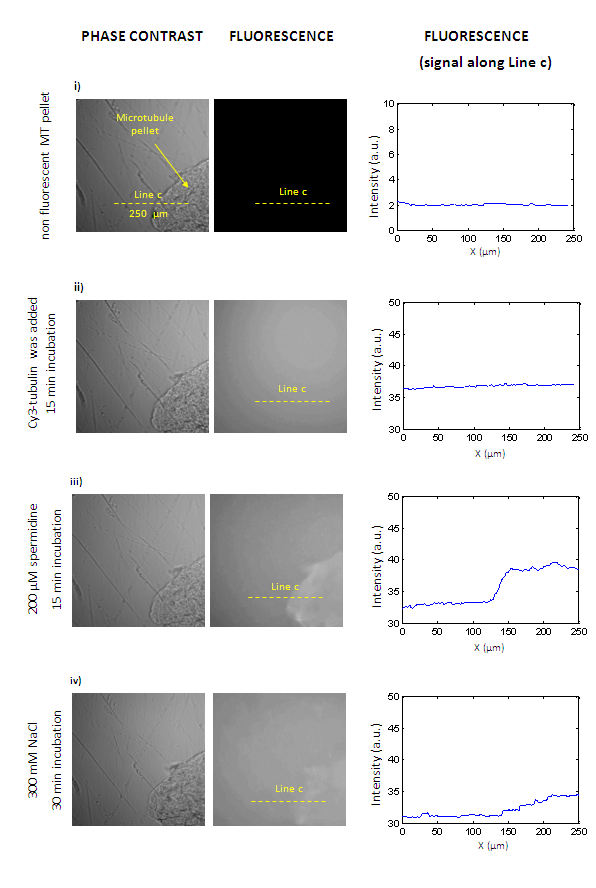

Supplement: Figure S1 — Attraction of fluorescent tubulin dimers onto microtubule mediated by spermidine. Fluorescence microscopy of a non fluorescent MT pellet in the presence of 0.1 µM Cy3-tubulin in buffer M without GTP. i) Microtubule pellet alone; ii) 0.1 µM of Cy3-tubulin was allowed to interact with the microtubule pellet for 15 min; iii) Addition of 200 µM spermidine (15 min); iv) Addition of 300 mM NaCl (30 min). Following the addition of spermidine, we observe the apparition of the microtubule pellet in the fluorescence image. The signal to background ratio due to Cy3-tubulin attraction in the pellet is about 13% under such condition, as shown in the line profile of fluorescent intensity. As expected for an electrostatic interaction, Cy3-tubulin was partly released from the microtubule pellet upon the addition of NaCl up to 300 mM. (0.16 MB TIF) [file pcbi.1000255.s001.tif]

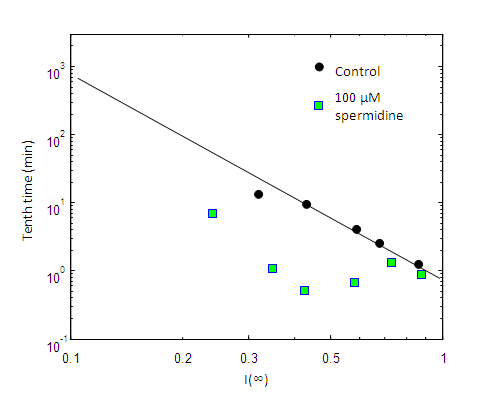

Supplement: Figure S2 — Log-log plot of the tenth time versus plateau value of the assembly curve extracted from Figure 7A. In the absence of polyamines, a straight line properly fits the experimental data. Its slope is about −3 in agreement with the results of Flyvbjerg et al [34]. This scaling properties is however not valid in the presence of spermidine. (0.02 MB TIF) [file pcbi.1000255.s002.tif]
