# Supplementary material for: Polyamine Sharing between Tubulin Dimers Favours Microtubule Nucleation and Elongation via Facilitated Diffusion
Source: PLoS Comput Biol. 2009 Jan 2;5(1):e1000255. doi: 10.1371/journal.pcbi.1000255 (PMC2599886; doi:10.1371/journal.pcbi.1000255)
Supplement: Text S1 — Attraction between tubulin dimers (0.05 MB DOC) [file pcbi.1000255.s003.doc]

**Attraction between tubulin heterodimer mediated by polyamine sharing**

To simplify the theoretical treatment, we will first consider that only multivalent cations are present in solution. We then use the Shklovskii theory [20], which is valid for mobile counterions (*here* *polyamines)* interacting non specifically with charged surfaces (*here* *tubulin*). In this simple and qualitative model, a new multivalent counterion coming on the tubulin surface repels the adsorbed ones, which then creates an image charge of opposite sign like on a metal surface. The attraction potential *ε* per ion at a distance *r* from the surface scales like $\left(\frac{Z^{2}}{r}\right)$ and it saturates for r=$\frac{1}{\sqrt{\pi n_{s}}}$ where *n_s_=σ/Ze* is the surface density of multivalent cations [20]. Therefore,$\frac{\varepsilon(n_{s})}{K_{B}T}\approx-Z^{2}\sqrt{\pi n_{s}}l_{B}$, where l_B_ is the Bjerrum length (*0.7nm*). In a further development, it can be shown that an attraction between two anionic surfaces can take place because there is an average energy gain *Δε* for multivalent cations to be localized at the interface between the two surfaces. *Δε* can be roughly estimated with a simple model assuming that the counterions at the tubulin:tubulin interface, prior to their overlapping, were adsorbed on the two surfaces according to their surface charge densities [22,24]:

$\Delta\varepsilon\approx\frac{\sigma_{1}}{\sigma_{1}+\sigma_{2}}\varepsilon(n_{s1})+\frac{\sigma_{2}}{\sigma_{1}+\sigma_{2}}\varepsilon(n_{s2})-\varepsilon(n_{s1}+n_{s2})$ (A1)

*σ_1_, σ_2_, n_s1_, n_s2_* are the surface charge densities and the multivalent counterion densities of the two surfaces (*1 and 2*). The energy gain *Δε* is particularly high when the two surfaces are highly negatively charged and have nearly the same surface charge density (*the best configuration occurs when σ_1_~_2_σ_2_*). As the surface charge densities of the C-terminal tail (*σ~1e^-^/nm²*) and the rest of tubulin body (*σ~0.1e^-^/nm²*) are very different, we have to consider the interactions between two C-terminal tails or between heterodimers that lack the C-terminal tails separately. For the interaction between heterodimers without the C-terminal tails, we can assume that *σ_1_ =σ_2_* and *Δε*  scales like:

$\frac{\Delta\varepsilon}{K_{B}T}\approx-Z^{2}\left(\sqrt{2\pi n_{s}}-\sqrt{\pi n_{s}}\right)\begin{matrix} \\ \end{matrix}l_{B}$ (A2)

For *σ=0.1 e^-^/nm²*, *Δε* is -0.16, -0.45, -0.84 and -1.3 *k_B_T* for Z=1 (*Na^+^,K^+^*); Z= 2 (*putrescine*), Z=3 (*spermidine*) and *Z*=4 (*spermine*) respectively. Therefore *Δε* is not very high even for higher valence multivalent cations like spermine. If we now assume that only *2Sσ/Ze* cations are implicated in the adsorption process, where *S* is the surface between the two interacting heterodimers, *U_el_,* the electrostatic energy gain due to the interaction between two tubulin dimers, equals *2Sσ/Ze×Δε* and may appear to be important, but the entropic loss due to the interpenetration of the clouds of counterions counterbalances this energy benefit. Indeed the entropic penalty in *K_B_T* unit for adsorbing one cation on a charged surface is *ln(c_s_ν_o_),* where *v_0_* is the molecular size of the counterions, and *c_s_* is the surface concentration of multivalent counterions and scales like *σ^3/2^*. Then the entropic cost in the association between two tubulin heterodimers is the sum of the energies required to insert counterions (*σ_1_/Ze*) and (*σ_2_/Ze*) into tubulin:tubulin interface (*here σ_1_ = σ_2_*). The thermal pressure of the interpenetrating clouds of counterion is [22]:

$\frac{\Delta E_{thermal}}{k_{B}T}\approx\frac{3}{2}S\left(\frac{\sigma_{1}}{Ze}\right)\ln\left(\frac{\sigma_{1}+\sigma_{2}}{\sigma_{1}}\right)+\frac{3}{2}S\left(\frac{\sigma_{2}}{Ze}\right)\ln\left(\frac{\sigma_{1}+\sigma_{2}}{\sigma_{2}}\right)$ (A3)

The total interaction energy, *U_C_,* is the sum of *U_el_* and *ΔE_thermal_*. For spermine, the most efficient cation to promote electrostatic attraction, we obtain that *Uc* per spermine ion is lower than -1 *K_B_T* (*-0.26 K_B_T*). Considering the thermal agitation, an attraction between tubulin dimers without the C-terminal tails can then hardly occur for natural polyamines. For spermidine, *Uc* per spermidine ion is positive (*repulsion*), 0.2 *K_B_T*.

Let us now consider the interaction between two C-terminal tails (*fig. 1*). The C-terminal tail of each tubulin monomer is roughly a line of charges with linear charge density *κ*. Its estimated value is situated around 5-10 e^-^/3.5 nm, which means that many negative sites are localized in the C-terminal regions compared to the rest of the molecule. After some straightforward mathematics, similar results to those obtained with the tubulin surface can be advanced for *Δε* but for a line of charges:

$\frac{\Delta\varepsilon}{K_{B}T}\approx-Z^{2}\frac{\kappa l_{B}}{Ze}$ (A4)

For κ=7e^-^/3.5nm, we obtain Δε~-1.4, -2.8, -4.2, -5.6 *K_B_T* for Z=1, 2, 3, 4 which is significantly larger than *K_B_T for z>1*. Considering a length of interaction *L_i_* of about 3.5 nm (*perfect match*) and that only half the counterions, *L_i_κ/Z,* are implicated in the adsorption mechanism (*see last paragraph*), thus *U_el_ ~-9.8 K_B_T* for *Z*= 1, 2, 3 and 4. For the entropic penalty, even if the geometry is different with the C-terminal tail, we will assume that equation (A3) is still valid. *2L_i_κ/Z* counterions are forced to be localized at the interface leading to *ΔE_thermal_* of about 14.5, 7.2, 4.8, 3.6 *K_B_T* for Z=1, 2, 3, 4. Therefore the total interaction energy, *U_C_= U_el_+ ΔE_thermal_,*, is 4.7, -2.6, -5 and -6.2 *K_B_T* for Z=1, 2, 3, 4, indicating that there is an attraction energy between two C-terminal tails for Z>1 which increases with the ion valence. We also remark that this interaction energy is significant for Z>1 compared to *K_B_T*. An interesting point is also to compare the energy benefit *U_C_* per polyamine ion to see if the correlations can really take place. For putrescine, spermidine and spermine, we obtain -0.37, -1.1 and -1.8 *K_B_T.* It indicates that correlations can hardly occur with divalent putrescine since the energy benefit is lower than *K_B_T* whereas they have a significantly higher chance to occur for spermidine and spermine.

In this analytical treatment and under such ideal conditions, we have however overestimated the energy benefit in counterion correlations by neglecting competition between monovalent. The replacement of multivalent counterions by monovalent ones reduces the number of multivalent cations participating in the counterion correlations. This effect is more pronounced for divalent putrescine than for higher valence cations (*see Text S2*). In addition, the electrostatic interactions among multivalent cations are generally screened at moderate and high ionic strengths.

Finally, it is worth noting that the total energy benefit per dimer results from the interaction of its two C-terminal tails and thus equals *2U_c_* (*fig. 1*).
