# Supplementary material for: Polyamine Sharing between Tubulin Dimers Favours Microtubule Nucleation and Elongation via Facilitated Diffusion
Source: PLoS Comput Biol. 2009 Jan 2;5(1):e1000255. doi: 10.1371/journal.pcbi.1000255 (PMC2599886; doi:10.1371/journal.pcbi.1000255)
Supplement: Text S2 — Competition between cations (0.03 MB DOC) [file pcbi.1000255.s004.doc]

**Effect of competition between monovalent cations and polyamines**

Let us assume that both nucleation and elongation rates are enhanced via facilitated diffusion triggered by polyamine sharing between tubulin dimers. The replacement of multivalent polyamines by monovalent counterions (*K^+^*) on tubulin surface decreases the energy gain in polyamine sharing and thus reduces the efficiency of facilitated diffusion. The binding ratio of polyamine ions (*η*) to one negative charge on the C-terminal tail surface depends on the monovalent salt concentration [25,29]:

$\eta=\frac{1}{Z}_{eff}\frac{\left[P^{Z+}\right]}{\left[KCl\right]^{Z_{eff}}}c_{s}^{Z_{eff}-1}\left(1-Z_{eff}\eta\right)^{Z_{eff}}$ (B1)

where *[P^Z+^]* is the polyamine concentration, *Z_eff_* is the effective charge of the polyamine which could be lower than the cation valence, *Z,* especially for linear polyamines like spermine and spermidine and *C_s_* is the concentration of the counterions on tubulin surface.

Upon increasing monovalent salt concentration, a sharp decrease of *η* is thus expected for polyamines, which occurs at lower concentrations of monovalent salt for putrescine than for spermidine or spermine. For example, with *C*s=6.7 M (*σ~1e^-^/nm²*) and 150 mM KCl, we obtain *Z_eff_* *η*≈0.5 for 300 µM spermidine (*Z_eff_ =3*) whereas *η* is significantly lower for 300 µM putrescine: *Z_eff_* *η*≈ 0.07 (*Z_eff_ =2*).

However the replacement of polyamines by monovalent cations can be compensated by an increase of polyamine concentration. Indeed, if $\frac{\left[P^{Z+}\right]}{\left[KCl\right]^{Z_{eff}}}$ is constant, then the polyamine binding ratio remains unchanged.
