# Supplementary material for: Polyamine Sharing between Tubulin Dimers Favours Microtubule Nucleation and Elongation via Facilitated Diffusion
Source: PLoS Comput Biol. 2009 Jan 2;5(1):e1000255. doi: 10.1371/journal.pcbi.1000255 (PMC2599886; doi:10.1371/journal.pcbi.1000255)
Supplement: Text S3 — Facilitated diffusion (0.04 MB DOC) [file pcbi.1000255.s005.doc]

**Facilitated diffusion to the MT ends**

The flow of tubulin arriving at one MT end scales like (*see equ. (1)*):

*J_facilitated_~ D_3_ C_free_ (πa²λ)^1/3^ ~2πD_2_ a C_s_/ λ* (C1)

Then, *λ* can then be extracted from these two equations:

$\lambda^{4/3}\approx\frac{2\pi^{2/3}D_{2}a^{1/3}C_{s}}{D_{3}C_{free}}$ (C2)

Assuming that many tubulins are able to adsorb on and to desorb from the MT surface, there is a statistical equilibrium between the concentration of adsorbed and free GTP-tubulin, which depends on the total attraction energy (*2Uc, two C-terminal tails per dimer)* [42]:

$\ln\left(\frac{\frac{C_{s}}{e}}{C_{free}}\right)=-2U_{C}/K_{B}T$ (C3)

where *e* is the thickness of the adsorption layer of tubulin (*arbitrary set to 4 nm*).

The total number of tubulin dimers is assumed to be constant in a volume v*,* ‘cell’ volume per MT of mean length *L*. The conservation of the number of tubulin molecules in a volume v gives:

$2\pi aC_{S}L+C_{free}v+nL\approx Cv$ with *2πaLρ<<*v (C4)

*C*v is the number of tubulin heterodimers in a volume v, *2πaC_s_L* is the number of tubulin adsorbed on MT, *C_free_*v is the free GTP-tubulin number, *nL* is the number of tubulin consumed in the MT (*n =1640 tubulins/µm*) and *ρ* is the thickness of the microtubule wall (*~ few nm*) . Using equ. C2, C3 and C4, some algebra yields:

$C_{S}=\frac{\left(Cv-nL\right)ye}{2\pi aLey+v}$ (C5)

$C_{free}=\frac{Cv-nL}{2\pi aLey+v}$ (C6)

where y=$e^{-2U_{C}/K_{B}T}$.

For low and moderate attractions, regime II, equ. C5 and C6 can be simplified: when $2\pi aLey<<v$, $C_{S}=\frac{\left(Cv-nL\right)ye}{v}$ and $C_{free}=\frac{Cv-nL}{v}$. For strong attractions*,* *regime III,* $2\pi aLey>>v$, thus: $C_{S}=\frac{\left(Cv-nL\right)ye}{2\pi aLey}$and $C_{free}=\frac{Cv-nL}{2\pi aLey}$. These equations were used to obtain J_facilitated_ for regimes II and III.
