# Supplementary material for: Polyamine Sharing between Tubulin Dimers Favours Microtubule Nucleation and Elongation via Facilitated Diffusion
Source: PLoS Comput Biol. 2009 Jan 2;5(1):e1000255. doi: 10.1371/journal.pcbi.1000255 (PMC2599886; doi:10.1371/journal.pcbi.1000255)
Supplement: Text S4 — Polymeric tubulin concentration (0.02 MB DOC) [file pcbi.1000255.s006.doc]

**Influence of facilitated elongation on polymeric tubulin concentration.**

To plot the polymeric tubulin concentration versus time (*fig. 9c*), *p(t)*, we assume that the number of microtubules, *N_m_*, is constant during assembly. It means that there is a fixed number of nucleus, *N_m_* at *t=0*. *p(t)* then scales like:

$p(t)\propto\int_{0}^{t} \left(J(t)-J_{0}\right)dt$ (D1)

where *J_0_* is the minimum flow of GTP tubulin to maintain elongation. According to Text 3 (*see also Regimes I, II and III in the elongation section*), *J(t)* depends on the independent variable *L(t); y, e, C,* v and *J_0_* being input parameters. To compute equ. *D1, L(t)*, the mean length of microtubule, is incremented proportionally, to *J(t)-J_0_*. At *t=0*, *L =12 nm* (*stable nucleus*). Let us note that v was related to *N* _m_ by considering that, if all tubulin is polymerized, the final length *L* will be *L_max_=10 µm*. We then obtain: *N_m_=C*v*/nL_max_*. The values of the input parameters are indicated in the legend of fig. 9.
